# Sparge Sampling of Molten Salts for Online Monitoring via Laser-Induced Breakdown Spectroscopy

**DOI:** 10.1021/acsomega.5c04988

**Published:** 2025-08-05

**Authors:** Zechariah B. Kitzhaber, Daniel Orea, Joanna McFarlane, Benjamin T. Manard, Hunter B. Andrews

**Affiliations:** † Radioisotope Science and Technology Division, 6146 Oak Ridge National Laboratory, 1 Bethel Valley Road, Oak Ridge, Tennessee 37830, United States; ‡ Nuclear Energy and Fuel Cycle Division, Oak Ridge National Laboratory, 1 Bethel Valley Road, Oak Ridge, Tennessee 37830, United States; § Chemical Sciences Division, Oak Ridge National Laboratory, 1 Bethel Valley Road, Oak Ridge, Tennessee 37830, United States

## Abstract

A method was developed to sample molten salts by sparging to generate and transport aerosols to an isolated instrument for compositional analysis by laser-induced breakdown spectroscopy (LIBS). Real-time monitoring of molten salt composition is critical to developing molten salt nuclear reactors, which offer enhanced safety and efficiency. In this article, the sparge sampling method is described and compared with sampling using a Collison nebulizer. The size distribution and transport of aerosols produced from molten eutectic NaNO_3_–KNO_3_ salt were compared for multiple gas flow rates (75–1200 mL min^–1^) and transport distances (0.68–2.61 m). Both methods produced aerosols ranging from 0.5 to 5.0 μm determined using a cascade impactor. Aerosols were effectively transported without pre- or trace-heating of gas lines, but transport efficiency was reduced by the formation of agglomerates. Sparge sampling was found to use less sample and less gas than a Collison nebulizer while producing a more concentrated aerosol stream (up to 5 μg L^–1^). The effects of laser energy and delay time on the signal quality of LIBS measurements of these aerosols were also studied. High energy and short delay times were found to enhance signal and repeatability, whereas signal-to-background and signal-to-noise ratios were highest at low energy and longer delay times. The capabilities of this system for online monitoring of molten salts were demonstrated with calibrations for Sr and Li with relative standard deviations of 2.6% and 1.5% and limits of detection of 380 and 180 μg g^–1^, respectively.

## Graphical abstract

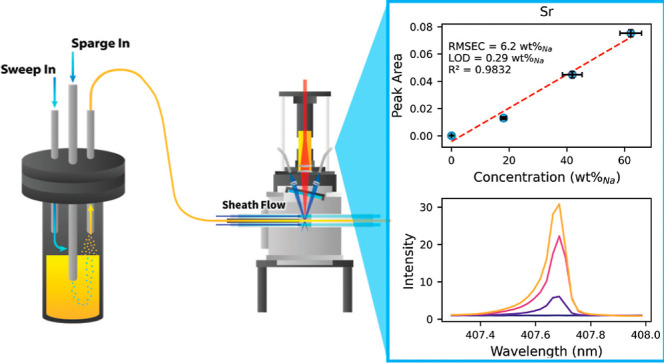

## Introduction

To meet the growing need for safe and efficient energy sources, many countries are turning to nuclear power, and advanced reactor designs are of particular interest. One category of advanced reactors that has received growing attention is molten salt reactors (MSRs). MSRs employ a high-temperature molten salt as the primary coolant, which improves the efficiency of electricity production and reduces risks associated with traditional reactors, including high pressures and the possibility of reactor meltdown.^1^ Some MSR designs also use a molten salt as liquid fuel by dissolving fissile material (e.g., UCl_3_, UF_4_) into the primary salt. In addition to their use in advanced reactor designs, molten salts are also used to electrochemically reprocess spent nuclear fuel and are being considered as a blanket material for fusion reactors to breed future fuel (e.g., tritium).^2^ Molten salts also have non-nuclear applications, including solar energy and chemical processing. Despite the growing interest in molten salts, there are barriers to their successful implementation in nuclear energy infrastructure. Namely, in situ analytical methods are needed to monitor the composition of molten salts and radionuclide transport in real time by tracking the ingrowth of transmuted elements, fission products, and contaminants from corrosion and ingression of air and moisture. In addition to monitoring the salt itself, the off-gas stream of an MSR must be monitored. This off-gas stream would include fission gases that are released into the headspace, other volatile species, and aerosols that are generated from the salt due to bubbling and agitation.^3^ Thus, the analytical methods must be able to handle a mixed-phase stream (i.e., gases and aerosols) that may be corrosive and contain radioactive material.

One technique that is well suited to handle the challenges of monitoring molten salt systems is laser-induced breakdown spectroscopy (LIBS). In LIBS, a high-powered pulsed laser is focused to a point on or within the sample; the coupling of the laser energy into the sample results in rapid heating, leading to ablation of the sample and formation of a microplasma containing excited atoms from the sample. As the plasma cools, these atoms emit photons with characteristic wavelengths. With sufficient spectrometer resolution, isotopes of both light and heavy elements can be differentiated, including isotopes of interest in MSRs—especially H and U.^4^ LIBS is a popular technique for elemental analysis of complex samples because it can detect elements from across the periodic table in a wide range of concentrations with little-to-no sample preparation. LIBS is compatible with solid, liquid, gas, or mixed-phase (aerosol) samples.^5,6^ These traits have made LIBS an attractive technique for in situ measurements in nuclear applications as well as a strong candidate for analysis of molten salts.^7,8^

Examples in literature demonstrate LIBS for analyzing molten salts, although many of these are proof-of-principle studies. Contaminants have been detected at micrograms per gram levels in frozen salt samples, but this is not amenable to real-time monitoring.^9–13^ Similar sensitivity has been achieved by directly firing the laser onto the surface of the molten salt; however, this resulted in issues associated with splashing, variable surface location relative to the optical focal point of the instrument, formation of oxidation layers on the surface, and plasma quenching.^14–19^ Due to the complications reported, aerosolization has been proposed as a potential real-time sampling approach. Previous studies have explored surrogate systems (i.e., aqueous aerosols and noble gases) to characterize the aerosols and demonstrate the capabilities of LIBS for monitoring aerosol-bearing off-gas streams.^20–24^ An aerosol sampling approach using a Collison nebulizer was successfully applied to sample molten LiCl–KCl eutectic spiked with U or Ce by Williams and Phongikaroon, who reported limits of detection (LODs) of 650 μg g^–1^ for U and 148 μg g^–1^ for Ce.^25–27^ More recently, a sparging approach was investigated by Andrews et al. that did not require a nebulizer, preheated gases, or trace heating between aerosol generation and LIBS measurement.^28^ However, optimization of the sampling parameters and evaluation of the quantitative capabilities of this approach are still needed. Also, because salt aerosols may differ in surface properties and other behavior from aqueous systems, characterization of aerosols produced from molten salts is needed.

A common method of generating aerosols is the Collison nebulizer. This device uses a fast-flowing gas stream passed over a narrow orifice to draw liquid through that orifice; the liquid is broken into small droplets as it mixes with the gas stream and is dispersed as a fine aerosol mist.^29^ An alternate method of generating aerosols is sparging. This method involves bubbling a gas through a liquid. As the bubbles reach the surface, they burst, releasing liquid droplets; larger droplets fall back into the liquid, but smaller droplets remain suspended in the headspace as aerosols. The size and number of aerosol droplets are affected by the size and number of bubbles, the surface tension and viscosity of the liquid, and the overall gas flow rate.^30^ The aerosols produced by sparging are of special interest in MSR development, as sparging may be used to remove fission gases from the fuel salt in an MSR.^3^

The present study builds upon the previous work from Andrews et al.^28^ and aims to optimize aerosol sampling of molten salts by (1) exploring sparging as an alternative aerosol-generating mechanism to a Collison nebulizer and characterizing the aerosol properties from both generation methods; (2) examining nonheated transport of aerosols at different distances and flow rates; and (3) developing demonstrative calibration models and assessing the LOD of LIBS for detecting contaminants in the sampled bulk salt. The investigated sampling approach eliminates the issues of maintaining a high temperature through the sampling line, allowing the aerosols to solidify as they are transported in a carrier gas to an isolated LIBS instrument. This technique is better suited to continuous online monitoring because it produces a more concentrated aerosol and reduces the total mass of aerosolized sample, gas consumption, and trace heating requirements. This reduction decreases cost and reduces the amount of radiation transport and the volume of associated waste streams. The aerosols produced by this method were characterized for a range of flow rates and transport distances and compared with aerosols produced using a Collision nebulizer. The size distribution and total mass of aerosol produced were determined using a cascade impactor and inductively coupled plasma-optical emission spectroscopy (ICP-OES). The analysis of these aerosols by LIBS is demonstrated in this article, and the optimal sampling conditions are identified. The LOD of this technique is presented for the total concentration of salt aerosols within a sample stream representative of an MSR off-gas, as well as for analytes within the bulk salt.

## Experimental Section

This study used a eutectic blend of NaNO_3_–KNO_3_ with a total mass of approximately 200 g. Although nitrate salts are more commonly used in solar power applications,^31^ they have been studied for use in molten salt reactors, and they were selected for this study because of their cost and stability, as salts more typical of an MSR (e.g., chlorides and fluorides) are air-sensitive and hygroscopic.

A schematic of the test setup is shown in Figure 1. This study used a salt vessel design similar to that described elsewhere.^28^ The salt vessel was constructed from a stainless steel chamber and sealed at the top with a flange with three ports: a sweep gas inlet, a sparge gas inlet through which a sparge tube could be inserted and submerged into the molten salt, and an outlet for the mixed gas/aerosol sample stream. The sweep gas inlet was not used in this study and to avoid confusion, is not shown in Figure 1. A thermocouple was inserted through the sparge tube to monitor the temperature within the salt vessel. The salt was contained in a glassy carbon crucible. The salt vessel was suspended in a benchtop programmable furnace set to 400 °C. The top flange was exposed outside the furnace, so it was lined with a heating trace set to 260 °C to limit the thermal gradient across the vessel. Temperatures within the salt vessel ranged from 260 to 300 °C—above the 223 °C melting point of the KNO_3_–NaNO_3_ eutectic salt. All equipment was placed inside a fume hood as a safety precaution against the possible release of aerosols or NO_*x*_ gases from the decomposition of the nitrate salts.^31^

**Figure 1:**
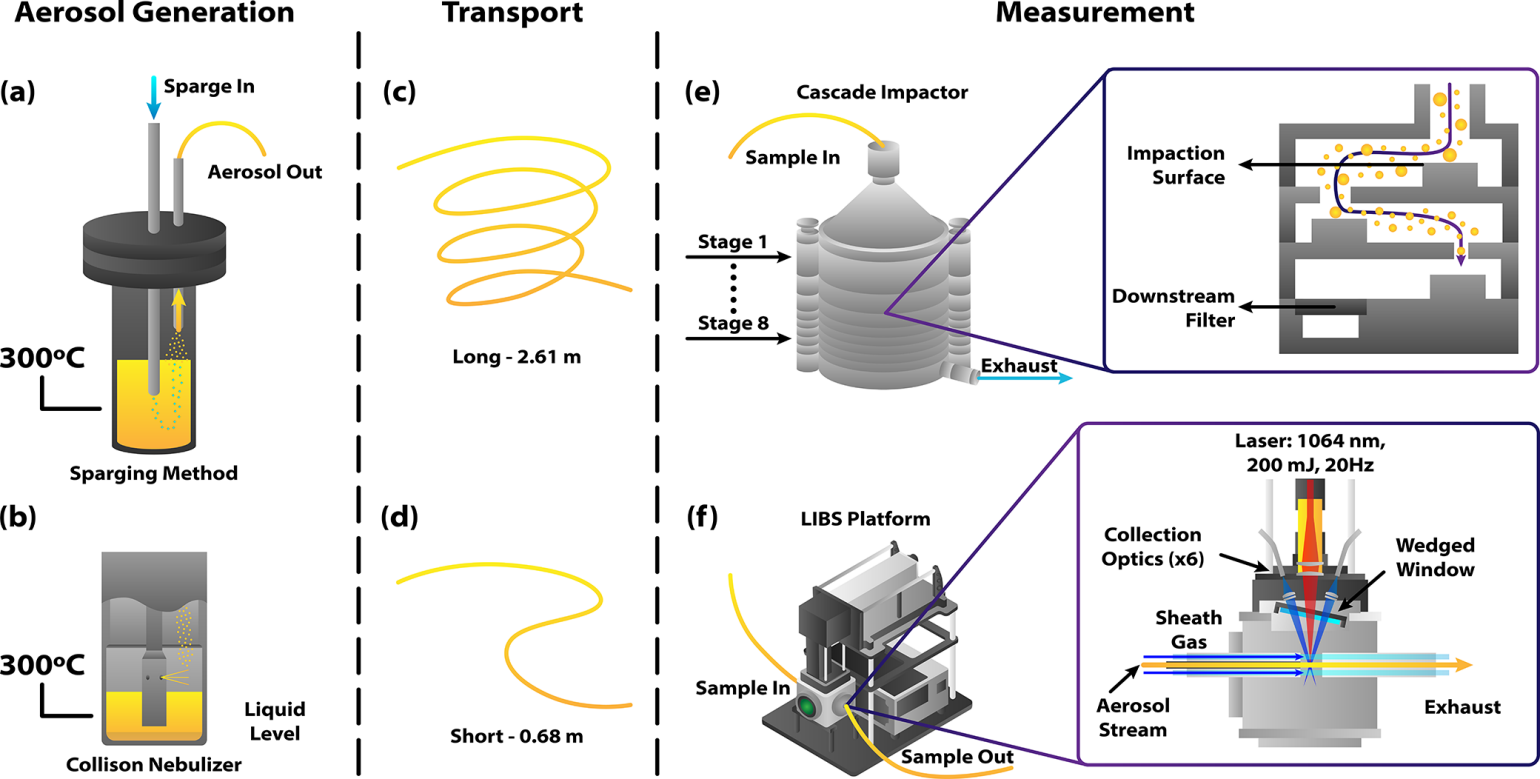
Diagram of experimental setup showing aerosol generation, transport, and measurement. The salt vessel was equipped with either (a) a sparging tube or (b) a Collison nebulizer. The generated aerosols were transported to the measurement point through (c,d) varying lengths of unheated polyethylene tubing. Lastly, the aerosols were measured using (e) a cascade impactor, which separates particles based on their size, or (f) a mobile LIBS platform. The LIBS measurement cell was equipped with a sheath gas to confine the sample stream and prevent coating of the optics.

Two methods of aerosol generation were compared: a sparge tube constructed from stainless steel tubing (3.18 mm inner diameter), and an in-house manufactured triple-jet Collison nebulizer (see Figure 1a,b). Argon gas (Airgas, 99.999%) was used both for sparging and as a sheath gas. The gas flow rate was measured using digital mass flow meters (Aalborg, GFM17). Aerosols were produced by flowing a gas through the sparge tube to either bubble it through the molten salt or to supply the Collison nebulizer depending on the experimental configuration. The resulting aerosols were almost instantly frozen (freezing time estimated to be < 0.01 s) in the nonheated (∼20 °C) gas stream and then transported from the outlet port to the cascade impactor or LIBS chamber through polyethylene tubing of varying lengths (see Figure 1c,d). Aerosols were collected in eight size fractions using a cascade impactor^32^ (TSI, Mini-MOUDI 135, see Figure 1e), then the impacted aerosols were analyzed by ICP-OES to determine the aerosol size distributions and generation rates. The LIBS instrument was a LIBS-8 (Applied Photonics, UK) equipped with a 1064 nm Nd:YAG nanosecond pulsed laser operated at 20 Hz (Litron, 200 Nano-MG) and a six-channel spectrometer with an integration time of 2 ms (Avantes, Avaspec 4096CL, see Figure 1f). The laser energy and delay time were varied, as discussed below. Additional details of sample preparation, measurement, and data analysis^33–38^ are available in the Supporting Information.

## Results and Discussion

### Aerosol Generation and Transport

The effectiveness of molten salt sampling by sparging was compared with a collison nebulizer. The aerosol size distribution and generation rate were determined from cascade impactor tests performed at a low and a high flow rate (300 and 1200 mL min^–1^) and at a short and a long aerosol transport length (0.68 and 2.61 m). The transport length represents the linear distance from the salt vessel outlet to the cascade impactor. Aerosols were considered to include particles from 0.32 to >10 μm in diameter, which were collected in seven size fractions on the calibrated stages of the cascade impactor. The size cutoffs for each stage represented the smallest aerosol collected at that stage. Aerosols smaller than 0.18 μm were not collected, and aerosols in the 0.18–0.32 μm range were below detectable levels in all trials. An additional size fraction representing aggregates and agglomerates (AA) was also collected before the first impactor stage, but these AA were excluded from the calculated aerosol concentrations. The results of these tests are shown in Table 1, arranged from highest to lowest aerosol concentration. Two of the eight trials, sparging at low flow with long transport and nebulization at a low flow rate with short transport, did not produce aerosols or AA at quantifiable levels (based on the ICP-OES method detection limit). In Table 1, "gen." refers to the method of aerosol generation, sparging ("Sparge") or Collison nebulizer ("Neb").

**Table 1:** Aerosol and AA Collected Masses and Estimated Aerosol Concentration

gen.	flow (mL min^–1^)	transport length	conc.^a^ (μg L^–1^)	total collected aerosol^a^ (μg hr^–1^)	collected AA (μg hr^–1^)
Sparge	300	short	8.86	159.5	7.72
Neb	1200	long	4.37	314	92
Sparge	1200	long	2.57	185	35.1
Neb	1200	short	2.14	154	2500
Neb	300	long	0.25	4.59	^b^
Sparge	1200	short	0.08	5.6^c^	9.7

a Does not include aggregates and agglomerates (AA).

b Below quantifiable levels.

c Uncertainty (RSD %) = ± 8%. All other uncertainties in this table are ±4%.

Figure 2 shows the size distribution of aerosols for the trials that produced the highest aerosol concentrations. Both the nebulizer and sparging produced aerosols with similar size distributions—mostly in the 1–3 μm range. These findings are consistent with aerosols produced by similar methods.^20,39^ However, these results are on the larger side of what was reported for molten salt aerosols in the Molten Salt Reactor Experiment and other molten salt aerosol tests, which reported aerosols in the nano- to micrometer size range.^40^

**Figure 2:**
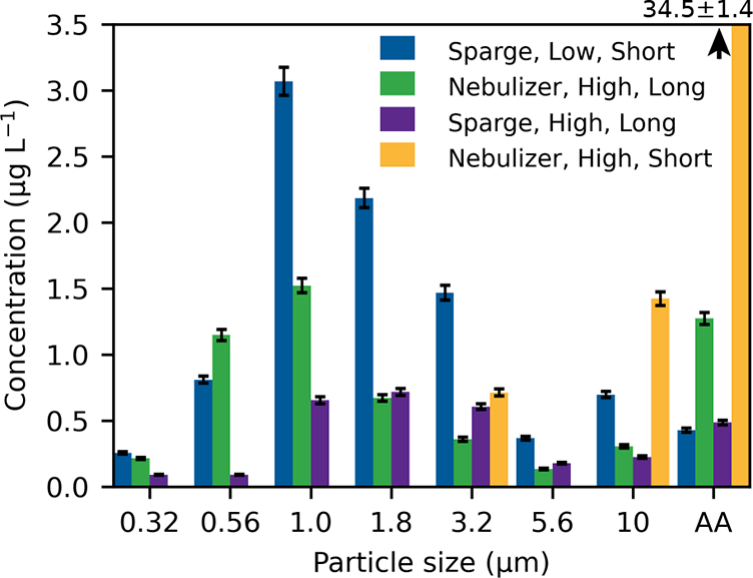
Aerosol size distribution plots from select trials. AA have been included in the plot, although they were not considered in the total aerosol calculations. The AA measured for the nebulizer at short distance and high flow rate are cut off in the plot; the measured value was 34.5 ± 1.4 μg L^–1^.

At the high flow rate, the nebulizer generated more aerosols than sparging. However, at the low flow rate, sparging was much more effective than the nebulizer and produced a higher aerosol concentration because of the increased dilution of the aerosol stream at the high flow rate. The highest overall aerosol concentration (8.86 μg L^–1^) was obtained by sparging at low flow. For the purposes of sampling an MSR, lower gas use and lower aerosol generation rates are desirable to minimize radiation transport and reduce operating cost from materials and maintenance. Additionally, AA formation is undesirable for molten salt sampling because transport efficiency is higher for smaller particles, and increased AA may contribute to clogging. This is evident in Table 1; fewer AA were observed at long transport. Flow rate may also influence AA formation and transport because more AA were observed at high flow rate. The nebulizer produced more AA than sparging; therefore, sparging at low flow is more amenable than the Collison nebulizer for continuously monitoring an MSR. For these reasons, a sparging flow rate of 300 mL min^–1^ was selectedfor LIBS optimization and calibration.

Transport distance and flow rate were both varied by a factor of about four, resulting in three distinct average expected transport times. Short distance/high flow had the fastest time—approximately 1 s—based on average flow velocity. Long distance/low flow had the slowest time—about 15 s. Both long distance/high flow and short distance/low flow had a medium time of about 4 s. In the trials with a slow transport time, no AA were observed, and aerosols were only detected in the 0.56 μm size fraction. This result suggests that higher flow rates may be required to maintain transport efficiency over longer distances. The highest aerosol concentrations were observed in the trials with a medium transport time, suggesting that AA fell out of the sample stream before reaching the cascade impactor while aerosols remained. These were the only trials that produced detectable aerosols in all seven size fractions from 0.32 to >10 μm. For the faster transport trials, aerosols smaller than 3.2 μm were not observed, and a large increase in AA occurred, as shown in Figure 2. This result indicates insufficient time for AA to fall out of the sample stream, leading to large amounts of AA abruptly accumulating at the cascade impactor. This accumulation may have caused smaller aerosols to be collected with the AA or otherwise affected the flow through the cascade impactor. This result shows that the distance and duration of transport from aerosol generation to sampling can influence the observed aerosols, as can the act of measuring those aerosols. Therefore, further research is needed to better understand transport efficiency from the point of generation to the measurement point—particularly for deployment scenarios in MSRs in which the LIBS measurement unit would be at extended distances (e.g., >10 m).

The LIBS signal is proportional to the number of atoms of the analyte species within the plasma, therefore, for aerosol samples, signal is proportional to the total mass of aerosol, rather than the number of aerosol particles. In general, more massive particles will produce larger signals, leading to enhanced sensitivity. Aerosol transport efficiency depends on a number of factors, including particle size, flow rate, tube diameter, thermophoresis, and electrostatic effects.^41–43^ Based on the size distribution of aerosols observed in this study (see Figure 2), future systems should be designed to maximize the transport of aerosols in the 0.5–5 μm range. This will require further research to understand the effects of thermophoresis and electrostatic forces in this setup.

### Optimization of LIBS Measurements

Following the analysis of aerosol generation and transport effects, the LIBS parameters were optimized using a four-level full-factorial design of energy (50, 100, 150, and 200 mJ) and delay time (3, 5, 7, and 12 μs). The same length of tubing was used during the LIBS measurements as that for the short transport aerosol tests (0.68 m); however, the overall length was slightly longer because of the sample chamber’s sheath gas inlet (0.30 m).

To identify the best conditions for measuring salt aerosols, four metrics were considered: signal, signal-to-background ratio (SBR), signal-to-noise ratio (SNR), and relative standard deviation (RSD). SBR is a common metric for identifying the quality of LIBS spectra. SNR also indicates spectral quality and is related to LOD. RSD describes the reproducibility of replicate measurements, when the reciprocal (RSD^–1^) is considered, a larger value indicates better reproducibility.

Figure 3 depicts the trends in signal, SBR, SNR, and RSD^–1^ from a subset of the experiment space, highlighting the preferred conditions (200 mJ energy, 5 μs delay). Each metric has been normalized so that a score of 1 represents the best performance. The corresponding average LIBS spectral peaks for Na are also shown; the offset between spectra is due to background. The shaded areas around each spectrum represent one standard deviation. As shown in Figure 3a,b, SNR and SBR were highest at low energy and long delay; however, signal and RSD^–1^ were low at those conditions. The greatest increase in RSD^–1^ was between 150 and 200 mJ, with only a minor decrease in SBR and SNR. Similarly, delays of 7 and 5 μs enhanced SNR and SBR compared with 3 μs, but RSD^–1^ decreased. A slight preference was given to RSD^–1^ to preserve measurement repeatability. Therefore, an energy of 200 mJ and a delay of 5 μs were selected for the calibration to provide the best balance between signal, SBR, SNR, and RSD.

**Figure 3:**
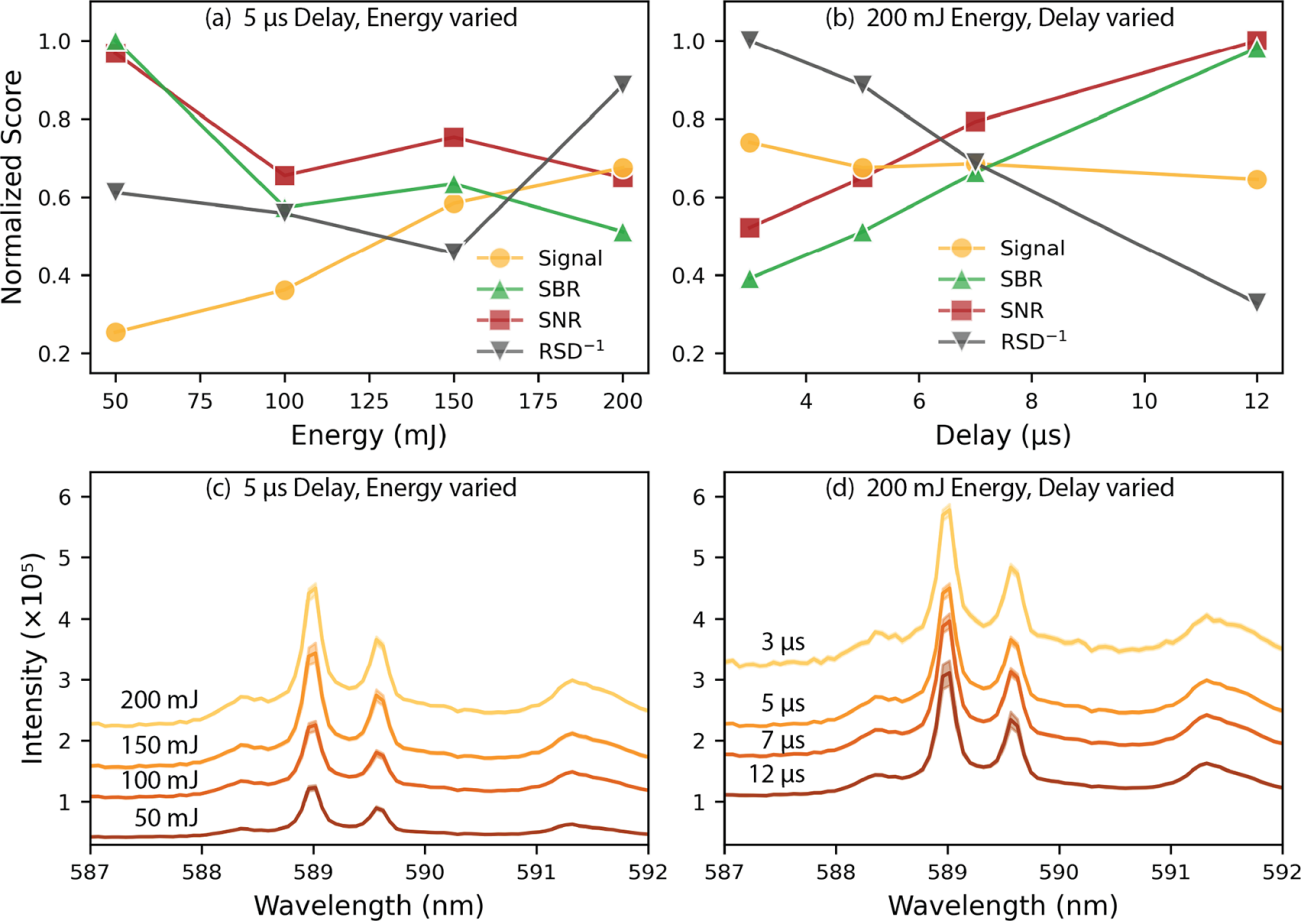
(a,b) Trends in signal, SBR, SNR, and RSD^–1^, and (c,d) spectral features for the Na doublet from a subset of the design space. RSD is plotted as the reciprocal so that for all four metrics, a score of 1 represents the best performance.

These results differ slightly from other studies, which have shown that the optimum SBR depends most strongly on laser energy and delay time, with a maximum at low energies and short delay times.^44–46^ The literature findings arise from the continuum background emission, which is greatest immediately following plasma formation and decays rapidly. Meanwhile, atomic signals increase during the early stages of plasma cooling before reaching a maximum. Surprisingly, in this study, no such local maximum in SBR was found. As expected, signal intensity increased with increasing laser energy and decreasing delay time. However, this increase in signal was accompanied by an even greater increase in background; therefore, the SBR followed an opposite trend. Thus, the conditions that maximized the SBR consequently minimized the signal. The likely cause of this behavior is the use of Ar as the carrier gas. LIBS experiments on solids under Ar gas streams have been shown to produce longer plasma lifetimes with slower decays.^47^ Given the large composition of Ar gas in the plasma volume, the range of delay times included in this experiment may not have been sufficient to maximize SBR. Therefore, future research will continue to explore the effects of various carrier gases (i.e., Ar and He) and the effect of flow rate on plasma properties.

Spectra were collected using the optimized LIBS parameters with sparging and nebulizing at 75–1200 mL min^–1^ and at 1500–2400 mL min^–1^ for nebulizing only. As previously noted, the same length of tubing was used during the LIBS measurements as for the short transport aerosol tests with the additional length of the sheath gas inlet. Figure 4 shows the observed LIBS signal based on the Na peak area at 589 nm for (a) each concentration measured at short transport and (b) each flow rate. Although only three of the short transport trials produced measurable aerosols, the LIBS signal was correlated with the measured aerosol concentration, as shown in Figure 4a. This result suggests that only aerosols—and not AA—were effectively transported to the LIBS at a short distance. As shown in Figure 4b, in the case of both sparging and the nebulizer, there was a cutoff flow below which aerosol concentration was too low to produce a LIBS signal. This cutoff appeared to be <75 mL min^–1^ for sparging and >600 mL min^–1^ for the nebulizer. Above this cutoff point, aerosol concentration was subject to two competing effects from generation and dilution. Increasing flow rate leads to an increase in the mass of aerosol generated but also has a dilution effect due to the increased volume of gas which reduces the aerosol concentration. At low flow rates, the generation effect is larger than the dilution effect, and there is a net increase in aerosol concentration, but at high flow rates dilution overcomes generation and overall concentration decreases. This results in a maximum in aerosol concentration at intermediate flow rates, consistent with results of similar experiments.^39^ This maximum appeared to be near 300 mL min^–1^ for sparging and around 1200 mL min^–1^ for the nebulizer. For all flow rates above the cutoff, Na was detectable by LIBS. The lowest measured aerosol concentration was 0.08 μg L^–1^, produced by sparging at 1200 mL min^–1^. The corresponding LIBS SNR for Na was 7.8 for a 100-shot averaged spectrum. A lower SNR of 2.5 was recorded for sparging at 75 mL min^–1^. Although the aerosol concentration was not measured for that trial, this result suggests that even lower aerosol concentrations are detectable.

**Figure 4:**
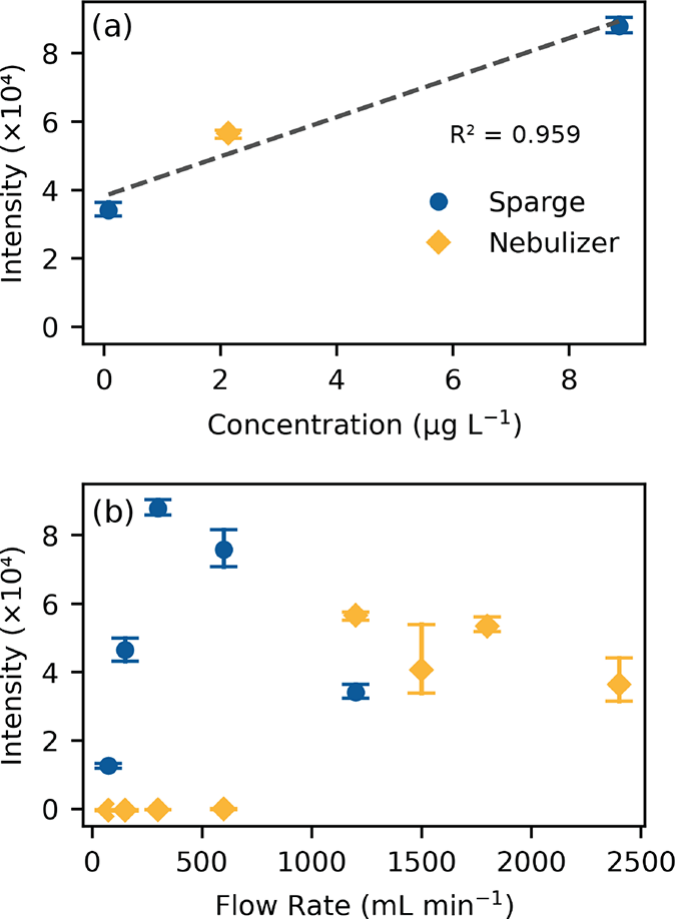
LIBS signal based on Na peak at 589 nm vs (a) measured concentration at short transport distances and (b) flow rate.

### Detection of Contaminants in the Bulk Salt

To demonstrate the feasibility of LIBS analysis for contaminants within the salt using this sparging approach, a calibration was performed using Sr and Li as representative analytes. These elements were chosen as analytes of interest because Sr is a common fission product and Li is a common surrogate for U isotopic studies and may be used as such in future studies. The salt was sparged with Ar using the conditions shown to produce the highest aerosol concentration and LIBS signal (sparging at 300 mL min^–1^). The LIBS settings for this calibration were 200 mJ pulse energy and 5 μs delay time—selected based on the results of the optimization to maximize signal and RSD^–1^. Figure 5 shows the average LIBS spectrum (in blue) of the calibration sample containing Sr and Li at 2.4 and 0.61 wt % in the bulk (wt_bulk_ %), respectively. The background (overlaid in red) was acquired by bypassing the salt vessel and flowing the sparge gas directly into the LIBS sample chamber to ensure no aerosols were present. Because Ar was the carrier gas, it was present at high concentrations, and the background can be mostly attributed to it, including the peaks from 735 to 850 nm, which are off the scale of the figure. Significant contributions also came from H, N, and O to the background likely because of residual moisture in the salt, the intrusion of atmosphere into the LIBS sample chamber, or impurities in the Ar gas. At high temperatures and in the presence of water, nitrate decomposes to NO_2_ gas.^31,48^ The salt temperatures used in this study should have resulted in minimal decomposition, but the presence of H in the LIBS spectrum suggests that moisture may have been present in the salt, leading to increased nitrate decomposition. Because of this decomposition, only a limited number of additions could be made, limiting the number of calibration samples. This decomposition also contributed to the presence of N and O in the background because of the accumulation of NO_2_ in the LIBS sample chamber. Chloride and fluoride salts are also sensitive to moisture, and it is well-understood that these salts need to be kept free from moisture and oxygen to avoid the formation of corrosive decomposition products. These observations both highlight the importance of maintaining salt purity, as well as demonstrate the capability of LIBS for monitoring contamination and decomposition species to confirm salt purity. One advantage of LIBS is that every element has multiple peaks to choose from, so while the stronger Ar lines are saturated, other lines are available with intensities that are more comparable with the other analytes. The present study used detectors with a low risk of damage due to saturation. To avoid damaging more sensitive or intensified detectors, it may be necessary to monitor only spectral regions that do not contain saturated peaks. Another advantage of LIBS is the ability to monitor a mixed sample stream of gases and aerosols. As seen in Figure 5, peaks representing elements at a wide range of concentrations in both gas and solid phases can easily be viewed simultaneously on a linear scale within a single spectral window of 400–700 nm.

**Figure 5:**
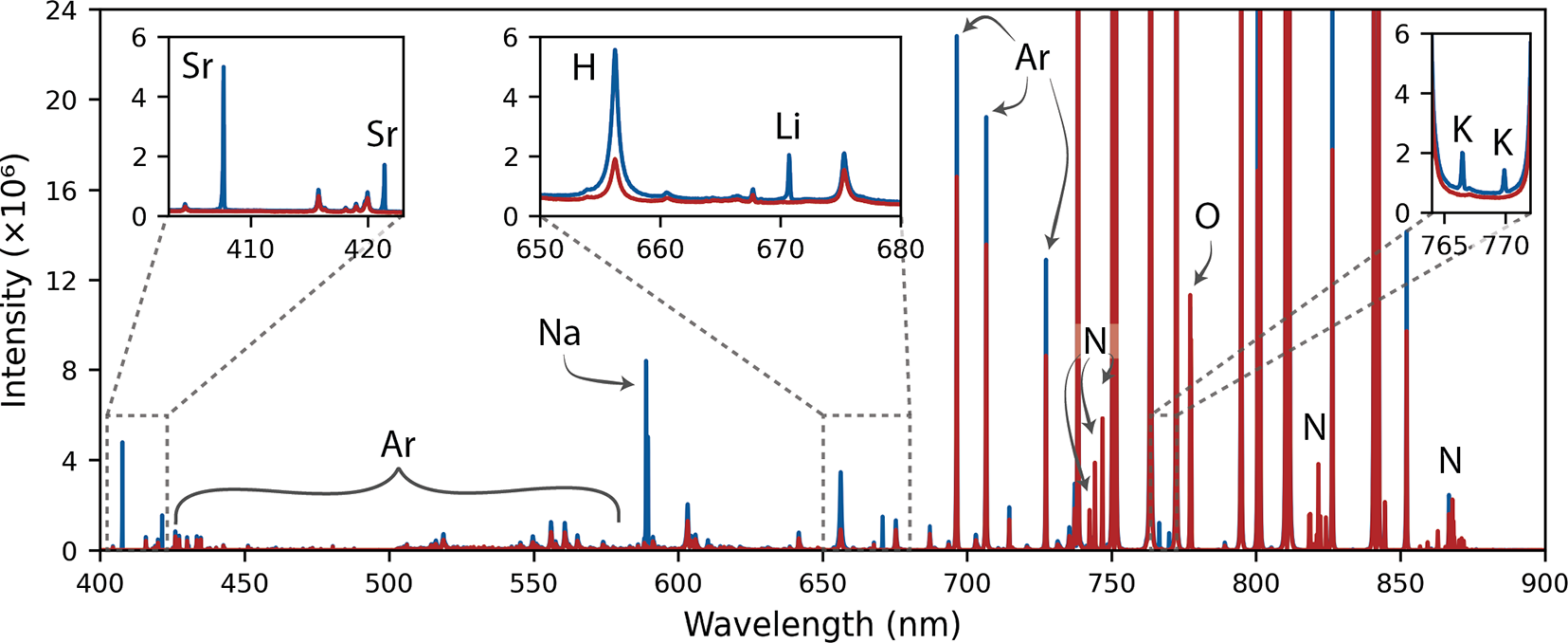
Representative LIBS spectrum that has been baseline-corrected using a rolling ball filter. The background, overlaid in red, is mostly made up of Ar, with notable contributions from H, N, and O because of intrusion of atmosphere, presence of moisture, and decomposition of nitrate to NO_2_ gas. Any unlabeled predominant peaks can be attributed to Ar, N, or O.

The calibration curves for Sr and Li are shown in Figure 6. For each calibration sample, concentrations of Sr, Li, Na, and K were determined using ICP-OES (the concentrations of N and O could not be determined). To reduce the effects of nitrate decomposition on sample concentration, the concentration values for the calibration were calculated as the weight percent relative to Na (wt %_Na_)—the element of the highest concentration for which ICP-OES data were available. For the same reason, LIBS spectra were normalized to the Na signal at 589 nm. This method also served to minimize the effect of fluctuations in aerosol concentrations in the gas stream. Despite these corrections, the quality of these calibrations was degraded because of the decomposition of nitrate—which made up over 65 wt % of the bulk salt—and the resulting uncertainty in sample concentration and limited number of samples. Due to these limitations, the data are insufficient for quantification, as indicated by the root-mean-square error of calibration (RMSEC), which represents how well the calibration model predicts the concentrations to which it was fit. The Li calibration exhibits behavior that may be due to self-absorption at higher concentrations, as indicated in Figure 6b, which would introduce nonlinearity which cannot be captured here due to the limited number of samples; however, with further calibration samples a higher-order fit may be used to account for this behavior. While the RMSEC is high, both models had LODs ≤ 0.3 wt %_Na_ and average RSD of analyte peak area <3%. A low RSD indicates precision, and an LOD well below the RMSEC indicates that the sensitivity and reproducibility of the method or technique are high, but the accuracy of the calibration data is low. The challenges presented by the decomposition of nitrate highlight two strengths of LIBS. First, both calibrations show acceptable *R* ^2^ and RMSEC despite changes in the matrix, indicating that LIBS is robust. Second, LIBS can confirm the decomposition of nitrate by detecting elevated levels of N and O in the background. This demonstrates that LIBS and aerosol sampling can be used to monitor for contamination and decomposition of salts. Performance is likely to improve in salts more relevant to MSRs (e.g., chlorides and fluorides), as these salts exhibit higher thermal stability and are therefore less prone to decompose, especially with increased efforts to reduce moisture and oxygen.

**Figure 6:**
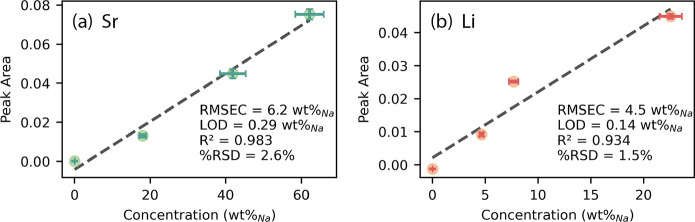
Calibration curves for (a) Sr at 407.7 nm and (b) Li at 670.8 nm. Error bars represent two standard deviations. The blank was below the ICP-OES method detection limit for Sr and Li, equivalent to 0.06 and 0.006 wt %_Na_, respectively.

Two methods were used to calculate the LOD. The LODs shown in Figure 6 were calculated according to eq 1, where σ_bl_ is the standard deviation of the net analyte signal for the blank, and *m* is the calibration slope.

1 $${LOD}_{SBL}=\frac{3\times \sigma_{bl}}{m}$$

While eq 1 is the form most commonly used and recognized throughout the analytical chemistry community, eq 2 is the recommended form in atomic emission spectroscopy (including LIBS) and it allows for a better estimate of the uncertainty in the LOD.^6,49^ eq 2 is a single point approach where SBR is the signal-to-background ratio, RSDB is the relative standard deviation in the background (expressed as a percent), and *c*_0_ is the analyte concentration for each point. In this case, both equations produced similar results.

2 $${LOD}_{SBR-RSDB}=0.03\times \frac{RSDB}{SBR}\times c_{0}$$

A single point LOD was calculated for each calibration sample using eq 2. The mean and standard deviation were 370 ± 200 μg g^–1^ for Sr and 140 ± 80 μg g^–1^ for Li, which include the LODs calculated using eq 1. Based on an aerosol concentration of 9 μg L^–1^, these values correspond to absolute concentrations in the sample stream of approximately 3 ng L^–1^ for Sr and 1 ng L ^–1^ for Li. These LODs are on par with or better than previously reported for LIBS analysis of molten salts. Although further experiments with additional samples are needed to verify these LODs, they confirm that the sensitivity and precision of LIBS are suitable for monitoring aerosol-bearing off-gas streams. It may be possible to improve the LOD by optimizing the LIBS parameters for individual analyte signals (e.g., Sr), rather than optimizing the bulk salt aerosol response (i.e., Na). Improvements in LOD have been reported using double-pulse LIBS and aerosol focusing techniques; future research should explore these modifications.^50,51^ Furthermore, an increase in the number of calibration samples and the use of multivariate methods would allow for corrections to self-absorption, leading to improvement in the LOD and other figures of merit.

## Conclusion

A sparge sampling method was demonstrated for the analysis of molten salt aerosols by LIBS. This approach produced a higher concentration of salt aerosols and consumed less salt and gas compared with a Collison nebulizer. This method serves to enhance analyte signal and reduce operating costs, transport of radioactive material, and waste generation. Although successful measurements of aerosols transported over distances up to2.6 m were demonstrated, aerosol transport efficiency was greatly reduced compared with shorter distances, and transport time was an important factor. In practice, much longer transport distances may be required to protect instrumentation from radiation effects, and a straight transport path may not be possible. Therefore, further research is needed to enhance the transport efficiency of aerosols between the salt vessel and LIBS measurement, with specific focus on thermophoretic and electrostatic effects. The sparge sampling approach offers equal or better LIBS LODs compared with other molten salt sampling methods without the requirement of preheated gases or trace-heated transport lines. The sparge tube design is low-cost and allows for fast replacement. All these considerations make sparging a more amenable approach to continuously monitoring an MSR. In addition to the usefulness of sparging as a method for sampling molten salts, the aerosols produced by this method are in a similar size range to what was reported in the molten salt reactor experiment and other molten salt experiments.^40^ Therefore, this system could be used to study aerosol transport and off-gas streams in MSRs. Future work will focus on enhancing accuracy and precision to improve LODs and applying this method to more MSR-relevant salts, including chloride and fluoride salts, which may exhibit different matrix effects. Improvements to the LIBS sample chamber to prevent the intrusion of atmosphere will be required to enable studying air-sensitive and hygroscopic salts, such as chlorides and fluorides. Other carrier gases, such as He, and improvements to aerosol transport efficiency and aerosol focusing techniques should be investigated in future work.

## Supplementary Material



## Data Availability

All relevant data that support these experimental findings are available from the corresponding author upon reasonable request.

## Notes

Additional details of sample preparation; cascade impactor, LIBS, and ICP-OES measurements; and data analysis; LIBS spectra of calibration samples (PDF)
