# Supplementary material for: Sparge Sampling of Molten Salts for Online Monitoring via Laser-Induced Breakdown Spectroscopy
Source: ACS Omega. 2025 Aug 5;10(33):37889–97. doi: 10.1021/acsomega.5c04988 (PMC12391992; doi:10.1021/acsomega.5c04988)
Supplement: Supplementary file 1 [file ao5c04988_si_001.pdf]

Supporting Information for:

Sparge Sampling of Molten Salts for Online Monitoring via Laser-Induced Breakdown Spectroscopy

Zechariah B. Kitzhaber[1], Daniel Orea[2], Joanna McFarlane[2], Benjamin T. Manard[3], Hunter B. Andrews[1*]

[1]Radioisotope Science and Technology Division, Oak Ridge National Laboratory, 1 Bethel Valley Road, Oak Ridge, TN 37830, USA (*email: andrewshb@ornl.gov)

[2]Nuclear Energy and Fuel Cycle Division, Oak Ridge National Laboratory, 1 Bethel Valley Road, Oak Ridge, TN 37830, USA

[3]Chemical Sciences Division, Oak Ridge National Laboratory, 1 Bethel Valley Road, Oak Ridge, TN 37830, USA

Aerosol measurements.

The aerosol size distributions were investigated using a cascade impactor (TSI, Mini-MOUDI 135, see Figure 1e). An aerosol-containing gas stream was produced using the nebulizer or by sparging the salt vessel with Ar. This gas stream was drawn through the cascade impactor using a pump (AP Buck Elite-5) operated at a flow rate of 2000 mL min^{-1}, which was required for accurate quantitation of particle size. A separate makeup inlet allowed filtered air in to compensate for the difference between the sample gas stream and cascade impactor pump flow rates. As the sample stream was drawn through the cascade impactor, aerosols were separated based on size; at each stage, aerosols larger than the cut-off size impacted the stage and were collected on a foil substrate, and smaller aerosols passed to the next stage.[32] This study used a cascade impactor with eight stages, collecting aerosols from 0.18 to >10 µm. An additional foil substrate was placed before the first stage to collect aggregates and agglomerates (AA). Aerosols were collected for 30–45 min, then each foil substrate was removed and placed in a vial. The impacted aerosols were dissolved in 5 mL of deionized water (18 MΩ·cm), then analyzed by ICP-OES to determine the concentration of K. Samples of the bulk salt were also analyzed to determine the mass fraction of K. The total mass of aerosol was calculated from these concentrations and the mass fraction, and the aerosol generation rate was calculated based on the collection time for each run. Aerosol concentration was estimated by dividing the generation rate by the flow rate for each trial.

LIBS measurements.

The LIBS instrument was a LIBS-8 (Applied Photonics, UK) equipped with a 1064 nm Nd:YAG nanosecond pulsed laser operated at 20 Hz (Litron, 200 Nano-MG) and a six-channel spectrometer (Avantes, Avaspec 4096CL, see Figure 1f). The laser was fired downward into the sample cell through a beam expansion module to minimize the beam waist at the focal point and through an angled window to prevent back reflections. The plasma emissions were collected by an array of optics off-axis from the

excitation laser. An integration time of 2 ms was used. The LIBS measurement cell was equipped with customized inlet and outlet flanges. A sheath gas was flowed concentrically around the sample stream at the inlet to prevent aerosols from depositing within the LIBS chamber or coating the optics.[23, 24] The sample chamber outlet was connected to a HEPA filter vacuum exhaust system to remove aerosols. The filtered exhaust was released into the fume hood.

Sample preparation.

All chemicals were purchased from Sigma Aldrich (99.9%, St. Louis, Missouri) and used as received. This study used a eutectic blend of $NaNO_3$–KNO_3 with a total mass of approximately 200 g. MSRs typically use chloride or fluoride salts, which are air-sensitive and hygroscopic. Although nitrate salts are not a common material in MSRs, they are used in solar power applications,[31] and they were selected for this study because of their cost and stability, as salts more typical of an MSR (e.g. chlorides and fluorides) are air sensitive. To dry the salt before the initial melting, the mixture was held at 120 °C for 24 h. Then, the mixture was gradually melted by increasing the furnace set point from 200 to 400 °C at a rate of 0.5 °C min^{-1}. The depth of the salt within the crucible was 3.5 cm. The salt remained molten and under inert conditions for the duration of the experiments (35 days). After measuring a blank of $NaNO_3$–KNO_3 eutectic, three serial additions of Sr and Li (as nitrates) were made. After each addition, the salt was allowed to homogenize for about 30 min before sparging with Ar at 300 mL min^{-1}. Five 1000 shot accumulate LIBS spectra were recorded for each calibration measurement. Spectra were recorded at least 10 min after sparging was started to ensure signal stability. Grab samples of the bulk salt were collected by dipping a glass rod into the molten salt; the layer of frozen salt that formed on the rod was then dissolved in deionized water and analyzed by ICP-OES.

ICP-OES measurements

Baseline trace element measurements were conducted via inductively coupled plasma – optical emission spectroscopy (ICP-OES). Here, an iCAP PRO (Thermo Scientific, Bremen, Germany) was employed such that axial viewing of the plasma was utilized. All samples were introduced to the ICP-OES via an autosampler (Elemental Scientific Inc, Omaha, NE, USA) via peristaltic pumping, into a quartz nebulizer housed within a quartz spray chamber. The measured samples were quantified via external calibration standards with standards (multi-element, High Purity Standards, Charleston, SC, USA) ranging from 0.1-10 µg mL-1.

Data Analysis.

Peaks were selected for the analysis of each element and were confirmed with the NIST Atomic Spectra Database.[33] Signal was calculated as the background-subtracted peak area using a Simpson integral. Background and noise were calculated individually for each peak by selecting a portion of the baseline within 200 points of the given peak consisting of 100 continuous points (covering a few nanometers) and containing no other peaks. The background was taken as the average in this region, and the noise was taken as the standard deviation. The signal-to-background ratio (SBR), signal-to-noise ratio (SNR), relative standard deviation of the signal (RSD), and LOD were calculated according to the equations given by Hahn et al.[6] All calculations were performed using Python (v 3.7.12)[34] with NumPy (1.26.4),[35] Pandas (2.2.2),[36,37] and SciPy (1.13.1)[38].

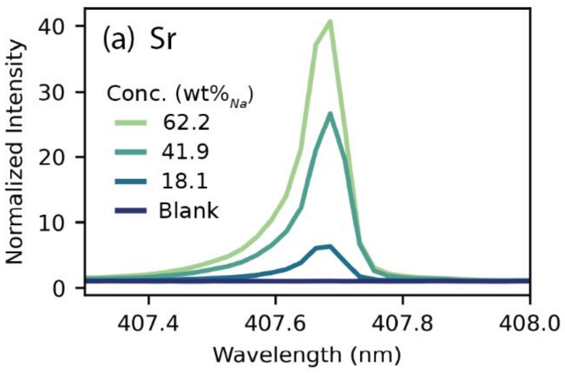

Figure S1. LIBS spectral peaks for (a) Sr and (b) Li from calibration samples.

References

(1) Serp, J.; Allibert, M.; Beneš, O.; Delpech, S.; Feynberg, O.; Ghetta, V.; Heuer, D.; Holcomb, D.; Ignatiev, V.; Kloosterman, J. L.; et al. The molten salt reactor (MSR) in generation IV: Overview and perspectives. *Progress in Nuclear Energy* **2014**, *77*, 308-319. DOI: 10.1016/j.pnucene.2014.02.014.

(2) Delpech, S.; Cabet, C.; Slim, C.; Picard, G. S. Molten fluorides for nuclear applications. *Materials Today* **2010**, *13* (12), 34-41. DOI: 10.1016/s1369-7021(10)70222-4.

(3) Andrews, H. B.; McFarlane, J.; Chapel, A. S.; Ezell, N. D. B.; Holcomb, D. E.; de Wet, D.; Greenwood, M. S.; Myhre, K. G.; Bryan, S. A.; Lines, A.; et al. Review of molten salt reactor off-gas management considerations. *Nuclear Engineering and Design* **2021**, *385*. DOI: 10.1016/j.nucengdes.2021.111529.

(4) Doucet, F. R.; Lithgow, G.; Kosierb, R.; Bouchard, P.; Sabsabi, M. Determination of isotope ratios using Laser-Induced Breakdown Spectroscopy in ambient air at atmospheric pressure for nuclear forensics. *Journal of Analytical Atomic Spectrometry* **2011**, *26* (3). DOI: 10.1039/c0ja00199f.

(5) Hahn, D. W.; Omenetto, N. Laser-Induced Breakdown Spectroscopy (LIBS), Part I: Review of Basic Diagnostics and Plasma-Particle Interactions: Still-Challenging Issues Within the Analytical Plasma Community. *Applied Spectroscopy* **2010**, *64* (12), 335A-366A. DOI: 10.1366/000370210793561691.

(6) Hahn, D. W.; Omenetto, N. Laser-induced breakdown spectroscopy (LIBS), part II: review of instrumental and methodological approaches to material analysis and applications to different fields. *Appl Spectrosc* **2012**, *66* (4), 347-419. DOI: 10.1366/11-06574.

(7) Kwapis, E. H.; Borrero, J.; Latty, K. S.; Andrews, H. B.; Phongikaroon, S. S.; Hartig, K. C. Laser Ablation Plasmas and Spectroscopy for Nuclear Applications. *Appl Spectrosc* **2024**, *78* (1), 9-55. DOI: 10.1177/00037028231211559.

(8) Cremers, D. A.; Beddingfield, A.; Smithwick, R.; Chinni, R. C.; Jones, C. R.; Beardsley, B.; Karch, L. Monitoring Uranium, Hydrogen, and Lithium and Their Isotopes Using a Compact Laser-Induced Breakdown Spectroscopy (LIBS) Probe and High-Resolution Spectrometer. *Applied Spectroscopy* **2012**, *66* (3), 250-261. DOI: 10.1366/11-06314.

(9) Hull, G.; Lambert, H.; Haroon, K.; Coffey, P.; Kerry, T.; McNaghten, E. D.; Sharrad, C. A.; Martin, P. Quantitative prediction of rare earth concentrations in salt matrices using laser-induced breakdown spectroscopy for application to molten salt reactors and pyroprocessing. *Journal of Analytical Atomic Spectrometry* **2021**, *36* (1), 92-102. DOI: 10.1039/d0ja00352b.

(10) Williams, A.; Bryce, K.; Phongikaroon, S. Measurement of Cerium and Gadolinium in Solid Lithium Chloride-Potassium Chloride Salt Using Laser-Induced Breakdown Spectroscopy (LIBS). *Applied Spectroscopy* **2017**, *71* (10), 2302-2312. DOI: 10.1177/0003702817709298.
(11) Sarkar, A.; Mukherjee, S.; Singh, M. Determination of the uranium elemental concentration in molten salt fuel using laser-induced breakdown spectroscopy with partial least squares-artificial neural network hybrid models. *Spectrochimica Acta Part B: Atomic Spectroscopy* **2022**, *187*. DOI: 10.1016/j.sab.2021.106329.
(12) Andrews, H.; Phongikaroon, S. Development of an Experimental Routine for Electrochemical and Laser-Induced Breakdown Spectroscopy Composition Measurements of SmCl3in LiCl-KCl Eutectic Salt Systems. *Nuclear Technology* **2018**, *205* (7), 891-904. DOI: 10.1080/00295450.2018.1551988.
(13) Andrews, H.; Phongikaroon, S. Electrochemical and Laser-Induced Breakdown Spectroscopy Signal Fusion for Detection of UCl-GdCl-MgCl in LiCl-KCl Molten Salt. *Nuclear Technology* **2021**, *207* (4), 617-626. DOI: 10.1080/00295450.2020.1776538.
(14) Effenberger Jr., A. J. Methods for Measurement of Heterogeneous Materials with Laser-Induced Breakdown Spectroscopy (LIBS). University of California, San Diego, 2009.
(15) Hanson, C.; Phongikaroon, S.; Scott, J. R. Temperature effect on laser-induced breakdown spectroscopy spectra of molten and solid salts. *Spectrochimica Acta Part B: Atomic Spectroscopy* **2014**, *97*, 79-85. DOI: 10.1016/j.sab.2014.04.012.
(16) Weisberg, A.; Lakis, R. E.; Simpson, M. F.; Horowitz, L.; Craparo, J. Measuring lanthanide concentrations in molten salt using laser-induced breakdown spectroscopy (LIBS). *Appl Spectrosc* **2014**, *68* (9), 937-948. DOI: 10.1366/13-07390.
(17) Lee, Y.; Foster, R. I.; Kim, H.; Choi, S. Machine learning-assisted laser-induced breakdown spectroscopy for monitoring molten salt compositions of small modular reactor fuel under varying laser focus positions. *Anal Chim Acta* **2023**, *1241*, 340804. DOI: 10.1016/j.aca.2023.340804.
(18) Lee, Y.; Foster, R. I.; Kim, H.; Garrett, L.; Morgan, B. W.; Burger, M.; Jovanovic, I.; Choi, S. Data Fusion of Acoustic and Optical Emission from Laser-Induced Plasma for In Situ Measurement of Rare Earth Elements in Molten LiCl-KCl. *Anal Chem* **2024**, *96* (28), 11255-11262. DOI: 10.1021/acs.analchem.4c00897.
(19) Lee, Y.; Yoon, S.; Kim, N.; Kang, D.; Kim, H.; Yang, W.; Burger, M.; Jovanovic, I.; Choi, S. In-situ measurement of Ce concentration in high-temperature molten salts using acoustic-assisted laser-induced breakdown spectroscopy with gas protective layer. *Nuclear Engineering and Technology* **2022**, *54* (12), 4431-4440. DOI: 10.1016/j.net.2022.07.014.
(20) Andrews, H.; McFarlane, J. *Characterization of Surrogate Molten Salt Reactor Aerosol Streams*; ORNL/TM-2021/2205; TRN: US2301935; Oak Ridge National Laboratory (ORNL), Oak Ridge, TN (United States), United States, 2021. https://www.osti.gov/biblio/1827008DOI: 10.2172/1827008.
(21) Andrews, H.; McFarlane, J.; Holcomb, D.; Ezell, D.; Myhre, K.; Lines, A.; Bryan, S.; Felmy, H. Sensor Technology for Molten Salt Reactor Off-Gas Systems. In 12th Nuclear Plant Instrumentation, Control and Human-Machine Interface Technologies (NPIC&HMIT 2021), 2021.
(22) Andrews, H. B.; McFarlane, J. Novel Calibration Approach for Monitoring Aerosol Hydrogen Isotopes Using Laser-Induced Breakdown Spectroscopy for Molten Salt Reactor Off-Gas Streams. *Sensors (Basel)* **2023**, *23* (24). DOI: 10.3390/s23249797.
(23) Andrews, H. B.; McFarlane, J.; Myhre, K. G. Monitoring Noble Gases (Xe and Kr) and Aerosols (Cs and Rb) in a Molten Salt Reactor Surrogate Off-Gas Stream Using Laser-Induced Breakdown Spectroscopy (LIBS). *Appl Spectrosc* **2022**, *76* (8), 988-997. DOI: 10.1177/00037028221088625.
(24) Andrews, H. B.; Myhre, K. G. Quantification of Lanthanides in a Molten Salt Reactor Surrogate Off-Gas Stream Using Laser-Induced Breakdown Spectroscopy. *Appl Spectrosc* **2022**, *76* (8), 877-886. DOI: 10.1177/00037028211070323.

(25) Williams, A.; Phongikaroon, S. Laser-Induced Breakdown Spectroscopy (LIBS) Measurement of Uranium in Molten Salt. *Applied Spectroscopy* **2018**, *72* (7), 1029-1039. DOI: 10.1177/0003702818760311.
(26) Williams, A. N.; Phongikaroon, S. Laser-Induced Breakdown Spectroscopy (LIBS) in a Novel Molten Salt Aerosol System. *Appl Spectrosc* **2017**, *71* (4), 744-749. DOI: 10.1177/0003702816648965.
(27) Williams, A. MEASUREMENT OF RARE EARTH AND URANIUM ELEMENTS USING LASER-INDUCED BREAKDOWN SPECTROSCOPY (LIBS) IN AN AEROSOL SYSTEM FOR NUCLEAR SAFEGUARDS APPLICATIONS. Dissertation, Virginia Commonwealth University, 2016.
(28) Andrews, H. B.; Kitzhaber, Z. B.; Orea, D.; McFarlane, J. Real-Time Elemental and Isotopic Measurements of Molten Salt Systems through Laser-Induced Breakdown Spectroscopy. *Journal of the American Chemical Society* **2024**. DOI: 10.1021/jacs.4c13684.
(29) May, K. R. The Collison Nebulizer: Description, Performance and Application. *Journal of Aerosol Science* **1973**, *4* (3), 235-238.

(30) Ginsberg, T. Aerosol Generation by Liquid Breakup Resulting from Sparging of Molten Pools of Corium by Gases Released During Core/Concrete Interactions. *Nuclear Science and Engineering* **1985**, *89* (1), 36-48. DOI: 10.13182/nse85-a17881.

(31) Wei, X.; Wang, Y.; Peng, Q.; Yang, J.; Yang, X.; Ding, J. NO x emissions and NO 2 - formation in thermal energy storage process of binary molten nitrate salts. *Energy* **2014**, *74*, 215-221. DOI: 10.1016/j.energy.2014.05.064.
(32) Rubow, K. L.; Marple, V. A.; Olin, J.; McCawley, M. A. A Personal Cascade Impactor: Design, Evaluation and Calibration. *American Industrial Hygiene Association Journal* **1987-6-1**, *48* (6). DOI: 10.1080/15298668791385174.
(33) Kramida, A.; Ralchenko, Y.; Reader, J., and NIST ASD Team. NIST Atomic Spectra Database. v5.12.; [Online]. Available: https://physics.nist.gov/asd [2025, February 21].National Institute of Standards and Technology: Gaithersburg, MD, 2024.
(34) *Python Language Reference*; Python Software Foundation.
(35) Harris, C. R.; Millman, K. J.; van der Walt, S. J.; Gommers, R.; Virtanen, P.; Cournapeau, D.; Wieser, E.; Taylor, J.; Berg, S.; Smith, N. J.; et al. Array programming with NumPy. *Nature* **2020**, *585* (7825), 357-362. DOI: 10.1038/s41586-020-2649-2 From NLM Medline.
(36) The pandas development team, *pandas-dev/pandas: Pandas*; Zenodo: 2020. https://doi.org/10.5281/zenodo.3509134 (accessed.
(37) McKinney, W. Data Structures for Statistical Computing in Python. *Python in Science* **2010**, 56-61. DOI: 10.25080/Majora-92bf1922-00a.
(38) Virtanen, P.; Gommers, R.; Oliphant, T. E.; Haberland, M.; Reddy, T.; Cournapeau, D.; Burovski, E.; Peterson, P.; Weckesser, W.; Bright, J.; et al. SciPy 1.0: fundamental algorithms for scientific computing in Python. *Nat Methods* **2020**, *17* (3), 261-272. DOI: 10.1038/s41592-019-0686-2 From NLM Medline.
(39) Mainelis, G.; Berry, D.; Reoun An, H.; Yao, M.; DeVoe, K.; Fennell, D. E.; Jaeger, R. Design and performance of a single-pass bubbling bioaerosol generator. *Atmospheric Environment* **2005**, *39* (19), 3521-3533. DOI: 10.1016/j.atmosenv.2005.02.043.
(40) Shahbazi, S.; Thomas, S.; Kam, D. H.; Grabaskas, D. *State of Knowledge on Aerosols and Bubble Transport for Mechanistic Source Term Analysis of Molten Salt Reactors*; 2022.
(41) Anand, N. K.; McFarland, A. R.; Kihm, K. D.; Wong, F. S. Optimization of Aerosol Penetration through Transport Lines. *Aerosol Science and Technology* **1992**, *16* (2), 105-112. DOI: 10.1080/02786829208959541.

(42) Chang, Y. C.; Ranade, M. B. A.; Gentry, J. W. THERMOPHORETIC DEPOSITION OF AEROSOL PARTICLES ON TRANSPORT TUBES. *Journal of Aerosol Science* **1990**, *21*.

(43) Liu, B. Y. H.; Pui, D. Y. H.; Rubow, K. L.; Szymanski, W. W. Electrostatic Effects in Aerosol Sampling and Filtration. *The Annals of Occupational Hygiene* **1985**, *29* (2), 251-269.

(44) Sirven, J.-B.; Mauchien, P.; Sallé, B. Analytical optimization of some parameters of a Laser-Induced Breakdown Spectroscopy experiment. *Spectrochimica Acta Part B: Atomic Spectroscopy* **2008**, *63* (10), 1077-1084. DOI: 10.1016/j.sab.2008.08.013.

(45) Yu, K.; Zhao, Y.; He, Y.; He, D. Response surface methodology for optimizing LIBS testing parameters: A case to conduct the elemental contents analysis in soil. *Chemometrics and Intelligent Laboratory Systems* **2019**, *195*. DOI: 10.1016/j.chemolab.2019.103891.

(46) Zheng, P.; Liu, H.; Wang, J.; Yu, B.; Zhang, B.; Yang, R.; Wang, X. Optimization of experimental conditions by orthogonal test design in a laser-induced breakdown experiment to analyze aluminum alloys. *Anal. Methods* **2014**, *6* (7), 2163-2169. DOI: 10.1039/c3ay41466c.

(47) Jõgi, I.; Ristkok, J.; Butikova, J.; Raud, J.; Paris, P. LIBS plasma in atmospheric pressure argon, nitrogen and helium: Spatio-temporal distribution of plume emission and Hα linewidth. *Nuclear Materials and Energy* **2023**, *37*. DOI: 10.1016/j.nme.2023.101543.

(48) Shanmugam Yuvaraj; Lin Fan-Yuan; Chang Tsong-Huei, a.; Yeh Chuin-Tih*. Thermal Decomposition of Metal Nitrates in Air and Hydrogen Environments. *The Journal of Physical Chemistry B* **2003**, *107* (4). DOI: 10.1021/jp026961c.

(49) Boumans, P. W. J. M. Detection Limits and Spectral Interferences in Atomic Emission Spectrometry. *Analytical Chemisty* **1994**, *66* (8), 459-467. DOI: 10.1021/ac00080a001.

(50) Windom, B. C.; Diwakar, P. K.; Hahn, D. W. Dual-pulse laser induced breakdown spectroscopy for analysis of gaseous and aerosol systems: Plasma-analyte interactions. *Spectrochimica Acta Part B-Atomic Spectroscopy* **2006**, *61* (7), 788-796. DOI: 10.1016/j.sab.2006.06.003.

(51) He, H.; Gao, Z.; Tian, H.; He, Y.; Liu, J.; Wang, Y.; Liu, Z.; Guo, B. Continuous emission monitoring the trace Sr from simulant aerosol emission with LIPS. *Spectrochimica Acta Part B: Atomic Spectroscopy* **2024**, *220*. DOI: 10.1016/j.sab.2024.107015.
